# Sophoridine derivative 6j inhibits liver cancer cell proliferation via ATF3 mediated ferroptosis

**DOI:** 10.1038/s41420-023-01597-6

**Published:** 2023-08-14

**Authors:** Kunpeng Tian, Jinrui Wei, Ru Wang, Mingming Wei, Fei Hou, Lichuan Wu

**Affiliations:** 1 School of Medicine, Guangxi University, Nanning, 530004 Guangxi PR China; 2 Pediatrics Research Institute of Hunan Province, Hunan Children’s Hospital, Changsha, 410007 Hunan PR China; 3 Guangxi Scientific Research Center of Traditional Chinese Medicine, Guangxi University of Chinese Medicine, Nanning, 530200 Guangxi PR China; 4 The State Key Laboratory of Medicinal Chemical Biology, College of Life Sciences, College of Pharmacy, Nankai University, Tianjin, 300071 PR China

**Keywords:** Liver cancer, Pharmacology

## Abstract

Liver cancer is one of the most lethal malignancies with an annual death of over 830,000 cases. Although targeted therapeutic drugs have achieved certain clinical efficacy, only sorafenib and lenvatinib are currently marketed as first-line targeted drugs to treat patients with advanced liver cancer. Therefore, developing more drugs are urgently needed. Ferroptosis is an iron-dependent programmed cell death (PCD) distinct from known PCDs including apoptosis, necrosis, and autophagy. Targeting ferroptosis is recognized as a promising potential therapeutic modality for liver cancer. Activating transcription factor 3 (ATF3) is an important ferroptosis inducer and targeting ATF3 offers a potential means to cancer therapy. In the present study, we reported for the first time a sophoridine derivative 6j with promising anti-liver cancer effects in vitro and in vivo. Compound 6j could induce liver cancer cells ferroptosis by promoting the accumulation of intracellular Fe^2+^, reactive oxygen species (ROS), and MDA. Inhibition of ferroptosis by ferrostatin-1 alleviated 6j induced accumulation of Fe^2+^, ROS, and MDA and restored cell viability. Further study revealed that compound 6j upregulated the expression of ATF3 via ER stress and knockdown of ATF3 by RNA interference attenuated 6j induced ferroptosis and cell proliferation inhibition. This study would provide new insights for the design of ferroptosis inducers and the development of anti-liver cancer drugs.

## Introduction

Liver cancer is the fourth most common cause of cancer related death, with an annual death of over 830,000 cases [1]. Hepatocellular carcinoma (HCC), intrahepatic cholangiocarcinoma (ICC), and HCC-ICC mixed type are the main types of liver cancer. Currently, the treatment of liver cancer mainly includes surgical treatment, radiotherapy and chemotherapy, targeted therapy, and immunotherapy [2]. Surgical treatment represents as the best strategy for liver cancer patients with early-stage. However, most patients are diagnosed in a late stage, losing the best opportunity for surgery. Systemic radiotherapy has greater toxicity and side effects, while traditional chemotherapy drugs (5-fluorouracil, cisplatin, doxorubicin and gemcitabine) have poor clinical effects [2]. Although targeted therapeutic drugs have achieved certain clinical efficacy, only sorafenib and lenvatinib are currently marketed as first-line targeted drugs to treat patients with advanced liver cancer, and patients are prone to drug resistance [2]. Immunotherapy has emerged as a promising treatment. However, the overall response rate of liver cancer patients to immunotherapy is less than 30% [3]. Even though the treatment of liver cancer has made great progress, problems such as drug resistance, few drugs available for patients to choose, and large side effects still represent as the main obstacles. Therefore, it is of great significance to develop new drugs for liver cancer.

Ferroptosis is a nonapoptotic necrotic cell death triggered by iron-dependent lipid peroxidation [4, 5]. As one of the organelles with the most abundant membrane lipids in cells, Endoplasmic reticulum (ER) is critical for the initiation of ferroptosis [6]. Factors such as lack of nutrition, hypoxia, imbalance of cellular calcium homeostasis, and chemotherapy could induce ER stress [7–9]. Substantial research displayed that ER stress could promote ferroptosis in various diseases [10–13]. ATF3, a transcription factor belongs to the ATF/CREB family, serves as a tumor suppressor in many tumors by inhibiting tumorigenesis, epithelial–mesenchymal transition, and tumor cell invasion and migration [14–16]. As a downstream target of ER stress pathway, ATF3 is up-regulated by PERK/ATF4 axis which is triggered by ER stress [17]. The activation of ATF3 is crucial for many chemotherapeutics induced ferroptosis, such as sorafenib [18], erastin [19], brucine [20], and cisplatin [21]. In recent years, ferroptosis has been reported to play a vital role in cancer treatment. It was found that ferroptosis can reduce the acquired resistance of tumor cells to anti-tumor drugs [22]. Induction of ferroptosis in tumor cells can enhance the effect of immunotherapy [23]. Ferroptosis is also revealed to mediate the synergy between radiotherapy and immunotherapy in cancer treatment [24]. These results suggest that ferroptosis is a potential target and drugging ATF3 would be an alternative for tumor therapy [25].

**Fig. 1:**
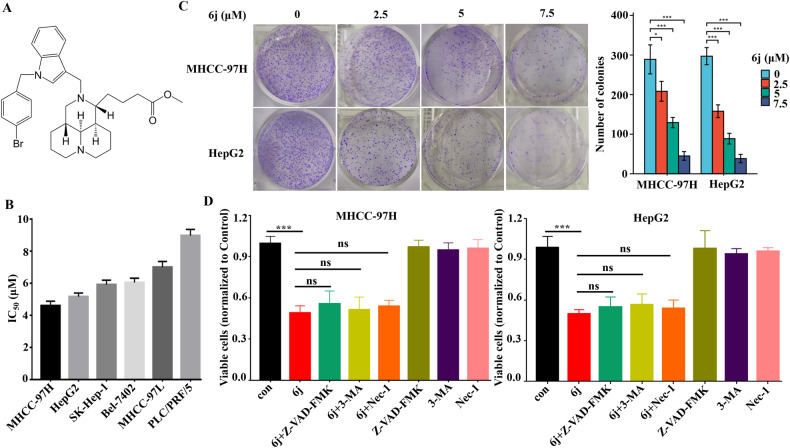
Compound 6j significantly inhibited liver cancer cell proliferation. **A** Structure of 6j. **B** IC50 of 6j against six liver cancer cell lines (*n* = 3). **C** Compound 6j suppressed colony formation of MHCC-97H and HepG2 cells in a dose dependent manner (*n* = 3). **D** Inhibitors of apoptosis, autophagy, and necrosis could not alleviate 6j induced cell proliferation inhibition (*n* = 3). *P < 0.05; **P < 0.01; ***P < 0.001.

Sophoridine, a natural plant alkaloid extracted from Sophora alopecuroides and the main active compound of the Chinese traditional medicine *FufangKushen injection* [26], was approved to cure patients with malignant trophoblastic tumors in 2005 [27]. It is well illustrated that sophoridine exerts its anticancer effects via inhibiting DNA Top I [28]. As an ideal lead compound, sophoridine displays multiple drug-like properties including special scaffold, simple structure, and high solubility [29]. In our previous study, series of sophoridine derivatives were synthesized and derivative 6j exhibited the strongest cytotoxic effects against liver cancer, lung cancer, and nasopharyngeal carcinoma without affecting the activity of Top I (Fig. 1A) [30], indicating that there might be an alternative anticancer mechanism for 6j. In the present study, the anti-liver cancer effects and mechanism of 6j was explored.

## Results

### Compound 6j inhibited liver cancer cell proliferation

First and foremost, the in vitro cytotoxic effects of 6j on six liver cancers were evaluated. It demonstrated that 6j exhibited a strong proliferation inhibition against these six liver cancer cell lines with IC_50_ below 10 μM (Fig. 1B). Notably, HepG2 and MHCC-97H cell lines were more sensitive than other four liver cancer cell lines. Thus, these two cell lines were selected for the next investigation. We then performed cell colony formation assays on these two cell lines which demonstrated that compound 6j significantly suppressed colony formation in a dose dependent manner (Fig. 1C). To reveal the action model of 6j induced proliferation inhibition, cells were co-cultured with 6j and inhibitors of apoptosis (Z-VAD-FMK), autophagy (3-MA), and necrosis (Nec-1) respectively following a cell viability measurement. The results displayed that Z-VAD-FMK, 3-MA, and Nec-1 had no impact on 6j induced cytotoxicity, indicating that 6j exhibited its antiproliferation effects via an alternative mechanism distinct from apoptosis, autophagy, and necrosis (Fig. 1D).

### Compound 6j induced ferroptosis in liver cancer cells

**Fig. 2:**
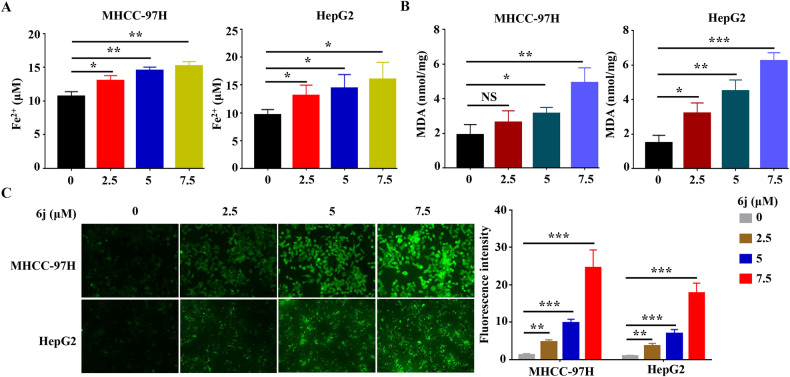
Compound 6j promoted liver cancer cell ferroptosis. Compound 6j induced accumulation of intracellular Fe^2+^ (**A**), MDA (**B**), and ROS (**C**) in a dose dependent manner (*n* = 3). *P < 0.05; **P < 0.01; ***P < 0.001.

Ferroptosis is a recently discovered new programmed cell death distinct from known cell death forms including apoptosis, autophagy, and necrosis [4], we then wondered whether 6j exerted its antiproliferation effects via inducing ferroptosis. To test this hypothesis, we firstly detected the content of intracellular Fe^2+^. The results showed that 6j could increase the intracellular content of Fe^2+^ in a dose dependent manner (Fig. 2A). Then, lipid peroxidation was evaluated and the results displayed that MDA, the indicator of lipid peroxidation was elevated in 6j treated cells (Fig. 2B). Besides, an obvious accumulation of ROS was observed in 6j treated cells (Fig. 2C). These results confirmed that 6j could induce ferroptosis in liver cancer cells.

### Ferroptosis inhibitor fer-1 could alleviate 6j induced cell proliferation inhibition

**Fig. 3:**
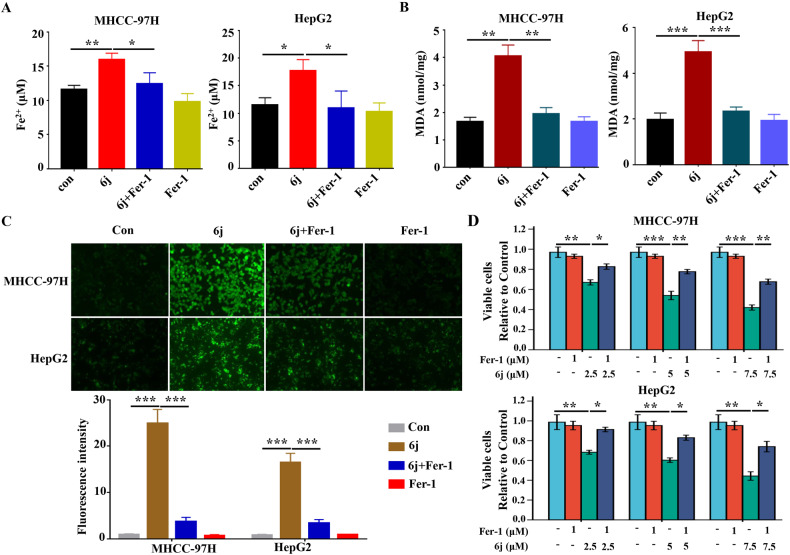
Ferroptosis inhibitor Fer-1 attenuated 6j induced ferroptosis and cell proliferation inhibition. Fer-1 treatment decreased 6j induced accumulation of Fe^2+^ (**A**), MDA (**B**), and ROS (**C**) (*n* = 3). **D** Fer-1 treatment alleviated 6j induced cell viability decreasing (*n* = 3). *P < 0.05; **P < 0.01; ***P < 0.001. All experiments were repeated three times independently.

To further validate the role of ferroptosis in 6j induced cell proliferation inhibition, cells were co-treated with ferroptosis inhibitor Fer-1 and 6j. Subsequently, the intracellular content of Fe^2+^, MDA, ROS and cell viability were measured. The results demonstrated that fer-1 could alleviate 6j induced accumulation of Fe^2+^, MDA, ROS (Fig. 3A–C) and cell proliferation inhibition (Fig. 3D). These results implied that 6j impeded liver cancer cell proliferation via inducing ferroptosis.

### Compound 6j activated ferroptosis-related genes and pathway

**Fig. 4:**
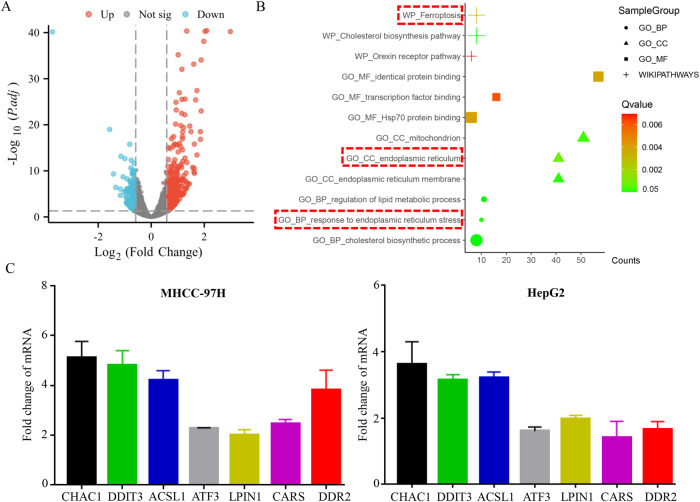
RNA-seq revealed that ferroptosis-related genes and pathway were activated upon 6j treatment. **A** Volcano plot of DEGs. **B** Pathway enrichment analysis of DEGs. **C** Validation of 7 ferroptosis-related genes via RT-qPCR (*n* = 3).

To elucidate the mechanism of 6j induced ferroptosis, RNA-seq was performed in 6j treated and non-treated cells. A total of 588 differentially expressed genes (DEGs) between 6j treated and non-treated cells were identified (Fig. 4A). Enrichment analysis of these 588 DEGs revealed that ferroptosis signaling pathway was significantly enriched (Fig. 4B). Then, 7 ferroptosis-related genes with most significant changes were chosen for validation by using RT-qPCR. Validation results demonstrated that all these seven genes were upregulated in 6j treated cells (Fig. 4C).

### Ferroptosis-related gene ATF3 is a potential target of liver cancer and might mediate 6j induced ferroptosis

**Fig. 5:**
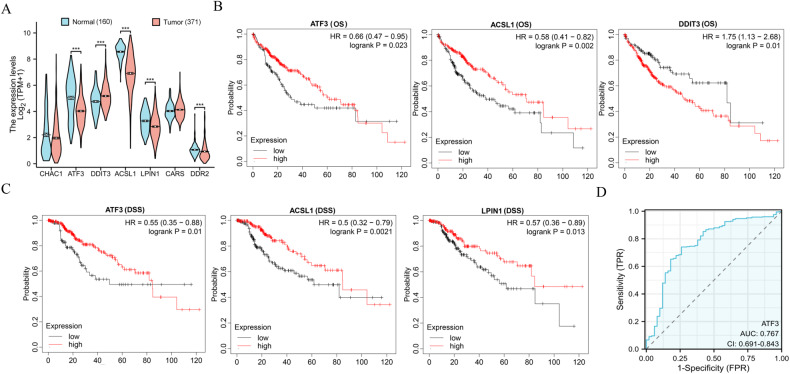
Expression and survival analysis identified ATF3 was a potential liver cancer target. **A** The expressions of 7 ferroptosis-related genes in liver cancer and normal tissues. The correlations between gene expression and OS (**B**) and DSS (**C**) of liver cancer patients. **D** ROC analysis of ATF3 in liver cancer via analyzing data from TCGA. *P < 0.05; **P < 0.01; ***P < 0.001.

To further reveal gene which mediated 6j induced ferroptosis, we first evaluated the expressions of these seven genes in liver cancer and normal tissues. By analyzing RNA-seq data from TCGA and GTEx, we discovered that ATF3, ACSL1, LPIN1, and DDR2 were significantly downregulated while DDIT3 was upregulated in liver tumor compared with normal tissues (Fig. 5A), implying that these five genes might be associated with liver cancer. Subsequently, we investigated the impacts of these five genes on OS and DSS of liver cancer patients. We found that patients with high expression of ATF3 and ACSL1 exhibited significantly longer OS while high expression of DDIT3 correlated with poor OS (Fig. 5B). The DSS analysis results displayed that liver cancer patients with high expression of ATF3, ACSL1, and LPIN1 exhibited significantly longer DSS (Fig. 5C). The above results suggested that ATF3 and ACSL1 might be correlated with liver cancer progression. Considering the results of RNA-seq that ATF3 showed a much more obvious changes than ACSL1 (FC = 1.34 for ATF3 and FC = 0.98 for ACSL1), we hypothesized that ATF3 might be the crucial gene which mediated 6j induced ferroptosis. Then, we performed ROC analysis to evaluate the diagnostic potency of ATF3 which demonstrated that ATF3 was a moderate predictor in liver cancer (AUC = 0.769, CI = 0.694–0.845) (Fig. 5D). These finding suggested that ATF3 was a potential target of liver cancer and might be the crucial gene mediating 6j induced ferroptosis.

### Compound 6j upregulated ATF3 expression via ER stress

**Fig. 6:**
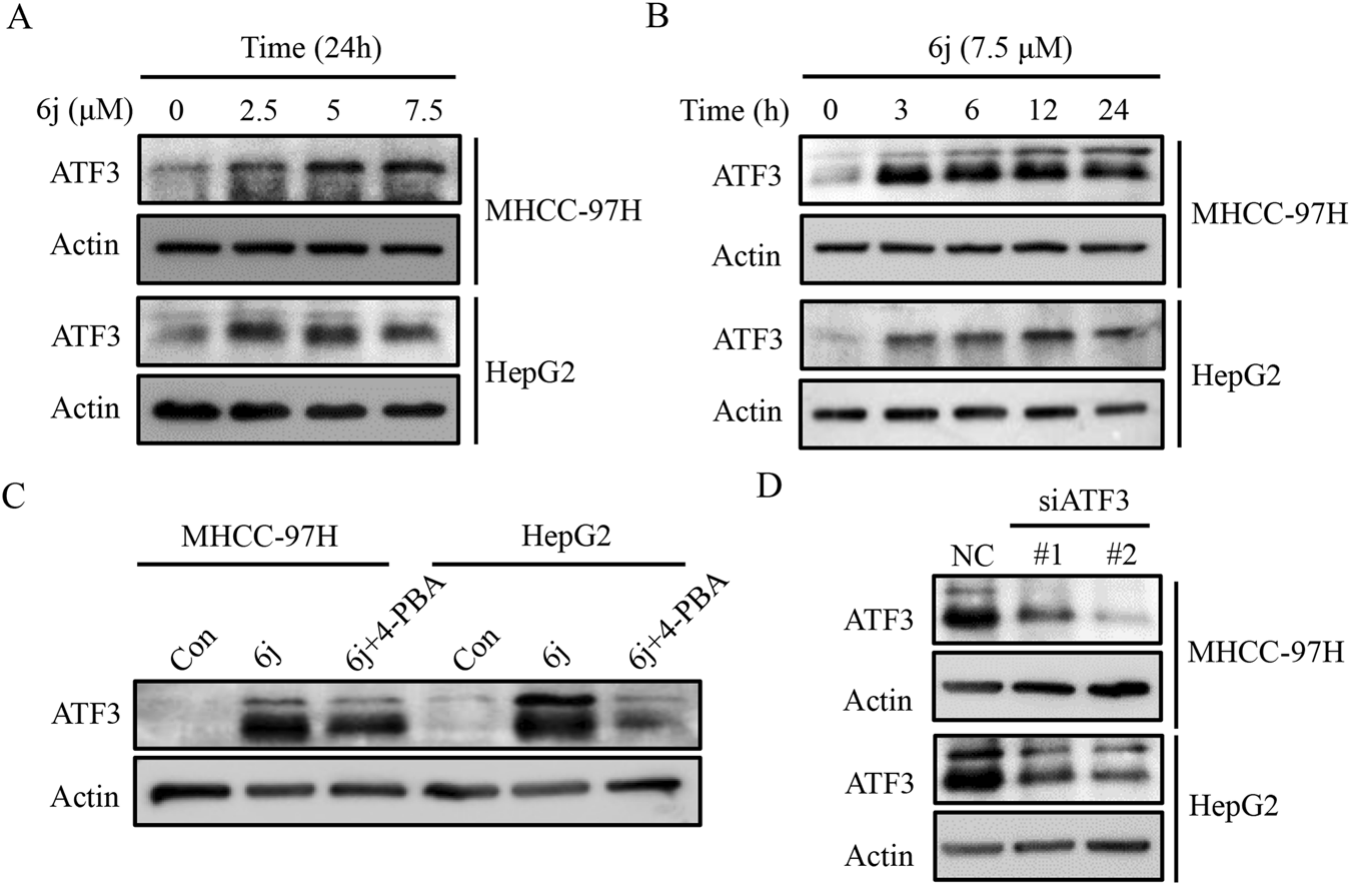
Compound 6j upregulated the expression of ATF3 via ER stress. **A** Compound 6j promoted the expression of ATF3 in a dose dependent manner in two liver cancer cell lines (*n* = 3). **B** Compound 6j promoted the expression of ATF3 in a time dependent manner in two liver cancer cell lines (*n* = 3). **C** ER stress inhibitor 4-PBA attenuated 6j induced ATF3 upregulation (*n* = 3). **D** RNAi efficiency detection by WB (*n* = 3).

Next, we evaluated the expression of ATF3 in liver cancer cells upon 6j treatment. The results demonstrated that ATF3 protein was significantly elevated by 6j in a dose and time dependent manner in both HepG2 and MHCC-97H cells (Fig. 6A, B). It has been reported that ATF3 is an important stress-associated protein and the activation of ER stress promoted the expression of ATF3 [31]. The RNA-seq and enrichment analysis identified that ER stress was activated (Fig. 4B), indicating that 6j might promoted the expression of ATF3 via ER stress. To verify, cells were co-treated with 6j and 4-PBA, an inhibitor of ER following a detection of ATF3 via WB. The results showed that inhibition of ER stress alleviated 6j induced upregulation of ATF3 (Fig. 6C). These results indicated that 6j induced ER stress contributed to the protein upregulation of ATF3.

### Compound 6j suppressed cell proliferation via ATF3 mediated ferroptosis

**Fig. 7:**
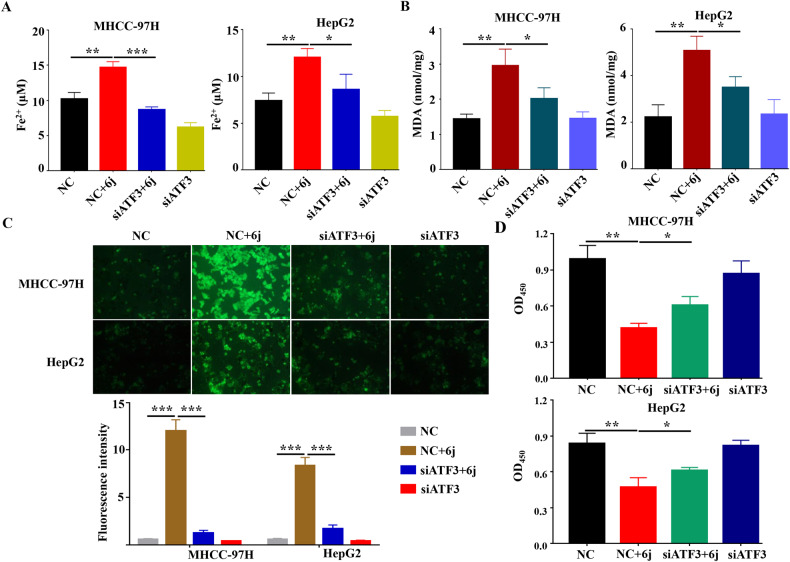
Knockdown of ATF3 attenuated 6j induced ferroptosis and cell proliferation inhibition. RNAi with ATF3 siRNA decreased 6j induced accumulation of Fe^2+^ (**A**), MDA (**B**), and ROS (**C**) (*n* = 3). D RNAi with ATF3 alleviated 6j induced cell viability decreasing (*n* = 3). *P < 0.05; **P < 0.01; ***P < 0.001.

Subsequently, we wondered whether the upregulation of ATF3 was responsible for 6j induced ferroptosis and cell proliferation inhibition. We first detected the RNAi efficiency in two HCC cell lines and found that siATF3 #2 conferred much higher efficiency than siATF3 #1 (Fig. 6D). Thus, siATF3 #2 was chosen for the subsequent assays. Cells were co-treated with siATF3 #2 and 6j and the intracellular content of Fe^2+^, MDA, ROS and cell viability were tested. We found that knockdown of ATF3 inhibited 6j induced ferroptosis by decreasing intracellular content of Fe^2+^, MDA, and ROS (Fig. 7A–C). Downregulation of ATF3 also alleviated 6j induced cell proliferation inhibition (Fig. 7D). These findings confirmed that ATF3 mediated 6j induced ferroptosis and the subsequent cell proliferation inhibition.

### Compound 6j exhibited promising anti-liver cancer effects in vivo

**Fig. 8:**
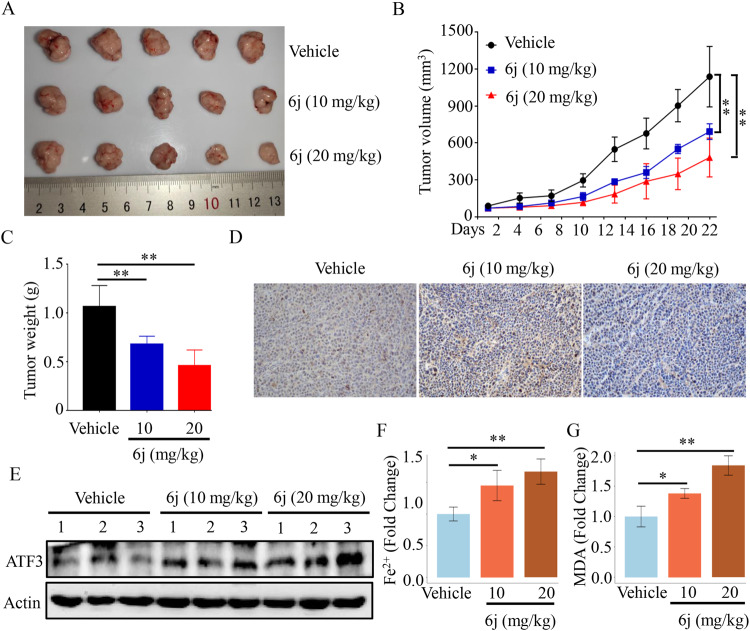
Compound 6j suppressed tumor growth. **A** Tumor Images after anatomy (*n* = 5 for each group). **B** Curves of tumor volumes at indicated times. **C** Statistic of tumor weight. **D** Representative expression image of Ki67 in tumor tissues from each group. **E** The expression of ATF3 in tumor tissues from each group. **F** Changes of Fe^2+^ in tumor tissues from different groups. **G** Changes of MDA in tumor tissues from different groups. *P < 0.05; **P < 0.01; ***P < 0.001.

We then assessed the in vivo anti-liver cancer effects of 6j via nude mice bearing HepG2 cells implanted subcutaneously into the right flank. Compound 6j was intraperitoneally injected twice a week. Compared with vehicle, tumor volume in low and high dose of 6j treatment groups were significantly reduced (Fig. 8A, B). Besides, significant tumor weight reduction was also observed in low and high dose of 6j treatment groups compared with vehicle treatment (Fig. 8C). IHC staining for Ki-67 revealed a pronounced reduction of Ki-67 in 6j treatment (Fig. 8D). We also found that 6j significantly increased the expression of ATF3 in vivo mouse xenograft in a dose dependent manner (Fig. 8E). The content of Fe^2+^ and MDA were also significantly increased in a dose dependent manner (Fig. 8F, G).

## Discussion

Cell death is closely related to the occurrence and development of tumors. In tumor cells, proteins that inhibit apoptosis (such as Mcl-1 and Bcl-2) are selectively up-regulated, while apoptosis promoting molecules (such as p53 and Bax) are mutated or downregulated, resulting in tumor cells insensitive to apoptosis [32]. From the perspective of morphology and mechanism, ferroptosis is different from apoptosis which is characterized by accumulation of intracellular Fe^2+^, ROS, and lipid peroxidation [4]. Own to the Warburg effect, tumor cells are characterized by elevated levels of ROS [33]. Thus, tumor cells are more sensitive to ferroptosis. Induction of ferroptosis in cancer cells is a promising strategy for cancer therapy [25]. A variety of drugs that have been used clinically or have strong clinical transformation potential can induce ferroptosis, such as sorafenib [34], lapatinib [35], zalcitabine [36], and neratinib [37]. Therefore, development of ferroptosis inducers is of great research significance and clinical value. In the present study, we reported a sophoridine derived compound 6j as a ferroptosis inducer in liver cancer cells. Compared with its parent compound sophoridine which showed an IC_50_ more than 5 mM (Figs. S1A), 6j displayed a significant anti-liver cancer effects with an IC_50_ below 10 μM. In mechanism, 6j exerted its anti-proliferation effects via inducing ferroptosis while ferroptosis plays no role in the sophoridine induced proliferation inhibition (Fig. S1B). Our study might shed the light on design of ferroptosis inducers based on sophoridine structure.

ATF3, a member of the ATF/CREB family of transcription factors [38], is widely expressed in human normal tissues including liver, gastrointestinal tract, and endocrine tissues (Fig. S2). Previous studies revealed that ATF3 could impede the tumorigenesis and progression of liver cancer [14, 39]. Meanwhile, ATF3 is reported to be a key mediator of drugs induced ferroptosis. Wang et al. found that ATF3 could enhance erastin-induced ferroptosis by suppressing system Xc [40]. Lu et al. identified that ATF3 mediated brucine-triggered glioma cell ferroptosis via increasing hydrogen peroxide and iron [20]. Fu et al. discovered that elevation of ATF3 could induce ferroptosis and alleviate cisplatin resistance in gastric cancer [21]. These results imply that ATF3 is a potential target of liver cancer and key mediator of ferroptosis. Developing ferroptosis inducers based on ATF3 has a good prospect. In the present study, we disclosed that a sophoridine derivative 6j could upregulated the expression of ATF3 via ER stress in liver cancer cell (Fig. 6). Knockdown of ATF3 abolished 6j induced ferroptosis and proliferation inhibition (Fig. 7). Our data further uncovered the role of ATF3 in ferroptosis and proved that ATF3 is a promising target for ferroptosis inducing drug design.

In aggregate, we reported a sophoridine derivative 6j which displayed a strong anti-liver cancer effect via ATF3 mediated ferroptosis. Our results suggest that upregulation of ATF3 by 6j could be a potential effective approach to treat liver cancer. There are also limitations to this work, including the mechanism of 6j induced ATF3 transcription activation, which remains to be further investigated.

## Materials and methods

### Reagents

Compound 6j was synthesized as reported previously [30]. Ferrostatin-1 (Fer-1, HY-100579), Z-VAD-FMK (HY-16658B), 3-Methyladenine (3-MA, HY-19312), Necrostatin-1 (Nec-1, HY-15760), and odium 4-phenylbutyrate (4-PBA, HY-A0281) were purchased from MedChem Express (New Jersey, USA). The Cell Counting Kit-8 (CCK-8, C0038), lipid peroxidation malondialdehyde (MDA, S0131M) assay kit, and Annexin V-FITC Apoptosis detection kit (C1062L) were obtained from Beyotime (Shanghai, China). Fe^2+^ (ADS-F-QT027) and ROS detection kits (ADS-W-G010) were purchased from Aidisheng (Jiangsu, China). The 2x SYBR Green real-time qPCR Kit (AG11701) was acquired from Agbio (Jiangsu, China). The small interfering RNA of ATF3 was purchased from GenePharma (Shanghai, China). The antibody against ATF3 (A1852) and Actin (4970S) were obtained from Abclonal(Wuhan, China) and Cell signaling technology(Boston, USA), respectively.

### Cell lines and culture

Six liver cancer cell lines (HepG2, PLC/PRF/5, MHCC-97H, MHCC-97L, Bel-7402, and SK-Hep-1) were purchased from NANJING COBIOER BIOSCIENCES CO., LTD and cultured in Dulbecco’s Modified Eagle Medium (DMEM) supplemented with 10% fetal bovine serum (FBS) and 1% penicillin-streptomycin. Cells in logarithmic growth phase were applied to experiments.

### Cell viability and colony formation assays

For cell viability assay, cells were plated into 96-well plate at a density of 5 × 10^3^ cells per well. Twenty-four hours later, cells were treated with different compounds for indicated time. Then, CCK-8 was added into each well and incubated for additional 2 h. Subsequently, the cell viability was measured by testing the absorbance at 450 nm. For colony formation assay, the HepG2 and MHCC-97H cells were seeded into 6-well plate with 800 cells per well. Then, cells were treated with different concentrations of 6j for about 7–10 days until the clonal populations were visible. Afterward, the cell clones were washed lightly with phosphate buffer solution (PBS) twice and fixed with methanol for 30 min. Subsequently, the clones were lightly washed with PBS twice again and stained with 0.05% crystal violet (IC0600, Solarbio, China) for 10–15 min. Finally, the clones were washed with PBS, dried, and photographed.

### Measurement of intracellular Fe^2+^, MDA, and ROS

HepG2 and MHCC-97H cells were seeded in 6-well plates at a density of 6 × 10^5^ cells per well. After 24 h, cells were treated with different concentrations of 6j for additional 16 h. Then, cells were collected and washed twice with PBS following the detection of intracellular Fe^2+^, ROS, and MDA according to the kit instruction, respectively.

### RNA interference (RNAi)

The HepG2 and MHCC-97H cells were plated into 6-well plates at a density of 2 × 10^5^ cells per well. Then, siRNA was mixed with Lipo3000 (L3000015, California, USA) at an optimum ratio of 1:1. After blending slightly, the mix was allowed to stand for 10–15 min at room temperature before being added to the plate well.

### RNA-seq and differentially expressed genes (DEGs) identifying

The total RNA was extracted from MHCC-97H cells treated with 6j (10 μM) or solvent control for 16 h. Subsequently, samples were sent to Biomarker Technologies (Beijing, China) for RNA sequence. DEGs were identified via applying DESeq2 with a criteria of |Fold change| >1.5 and *p* < 0.05. The RNA-seq data used in this study has been uploaded in the GEO dataset (GSE237138).

### Real-time qPCR (RT-qPCR)

After cells were treated with different concentrations of 6j, total RNA was exacted with TRIzol reagent (AG21102, agbio, China). Then, cDNA synthesis was carried out with EVO M-MLV RT MIX Kit (AG11706, agbio, China). RT-qPCR was performed by SYBR Green Pro Taq HS qPCR Kit according to reagent instructions. The primer pairs used for RT-qPCR were as follows: CHAC1-F: GTGGTGACGCTCCTTGAAGATC; CHAC1-R: GAAGGTGACCTCCTTGGTATCG; ATF3-F: ATGATGCTTCAACACC CAGGC; ATF3-R: TTAGCTCTGCAATGTTCCTTC; LPIN1-F: CCAGCCCAATGG AAACCTCC; LPIN1-R: AGGTGCATAGGGATAACTTCCTG; ACSL1-F: GACATT GGAAAATGGTTACCAAATG; ACSL1-R: GGCTCACTTCGCATGTAGATA; DDR2-F: CCAGTCAGTGGTCAGAGTCCA; DDR2-R: GGGTCCCCACCAGAGT GATAA; CARS-F: GGTGACGTGGTATTGCTGTG; CARS-R: CTCTTCTCCCGA TACTGCTCG; DDIT3-F: GGAAACAGAGTGGTCATTCCC; DDIT3-R: CTGCTT GAGCCGTTCATTCTC; GAPDH-F: ACAACTTTGGTATCGTGGAAGG; GAPDH-R: GCCATCACGCCACAGTTTC.

### Western blotting (WB)

Briefly, the HCC cells were treated with different drug concentrations. The samples were lysed with RIPA lysis buffer (R0278, sigma, China) containing proteinases inhibitor cocktail. Then, the protein concentration was measured by a BCA protein kit. After that, the protein was subjected to sodium dodecyl sulfate-polyacrylamide (SDS)-page and transferred to a polyvinylidene difluoride (PVDF) membrane. Subsequently, the membrane was blocked with 5% non-fat milk for 1 h and incubated with indicated primary antibodies overnight at 4 °C. Finally, the membranes were incubated with secondary antibody (111-035-003, Jackson ImmunoResearch, USA) for 1 h at room temperature and then scanned on iBright 1500 (Invitrogen, USA) with appropriate ECL (A38556, Thermofisher, USA).

### Immunohistochemistry (IHC)

The samples from xenograft mouse models were fixed with formalin and then embedded in paraffin. Afterward, paraffin-embedded tissue was cut into histological sections. Then sections were dewaxed in xylene and rehydrated with graded alcohol. Subsequently, the citrate buffer was applied to retrieve antigen at 100 °C, 10 min, and the sections were blocked with 5% BSA for 1 h and incubated with primary ki-67 antibody (ab16667, Abcam, UK) at 4 °C for 24 h. The appropriate secondary antibody was added to the sections at 37 °C for 90 min after the primary antibodies were washed twice with PBS. Then, sections were washed with PBS to remove the secondary antibodies and dripped with DAB solution for 10 min. Finally, the sections were stained with hematoxylin.

### In vivo mouse model

The animal studies were performed according to protocols approved by the Animal Ethics Committee of Guangxi University (GXU-2022-161). A subcutaneous tumor bearing mice model was constructed as reported previously after 5–10 min of perfusion with isoflurane with rodent anesthesia machine [41]. Briefly, 5–6-week-old female BALB/C nude mice were purchased from Charles River (Beijing, China) and HepG2 cells with 5 × 10^6^/ml were subcutaneously injected into the right flank. When tumor grown to a volume of about 50 mm^3^, mice were randomly divided into three groups (vehicle control, 10 mg/kg, and 20 mg/kg of 6j, *n* = 5). Mice were treated with indicated compounds twice a week for three weeks. Twenty-two days later, mice were euthanized and the tumor weight was measured, photographed, and fixed with paraformaldehyde. Tumor volume was calculated as the following formula: Volume = length × width^2^ × 0.5. No blinding was performed during the mouse experiments.

### Bioinformatic analysis

The different expressions of seven ferroptosis-related genes between liver tumor and normal tissues were evaluated via analyzing RNA-seq data from TCGA and GTEx. For survival analysis, the online database Kaplan-Meier (http://kmplot.com/analysis/) was used to investigate the impacts of seven ferroptosis-related genes on overall survival (OS) and disease special survival (DSS) of HCC patients. The time-related receiver operating characteristic curve (ROC) was performed to assess the OS predictive ability of ATF3 in HCC by running R package “pROC”.

### Statistical analysis

The SPSS 11.0 software was used for the analysis of the experimental results. The *T* test was used to compare the measurement data between the two groups. *P* values < 0.05 were considered statistically significant. All experiments were repeated at least three times and data are presented as the mean ± SD unless noted otherwise. *p < 0.05; **p < 0.01; ***p < 0.001.

### Supplementary information

Figugre legends for Supplementary

Figure S1

Figure S2

Full and uncropped western blots

## Data Availability

The data supporting the findings of the present study are available from the corresponding author upon reasonable request.
