# Supplementary material for: Sophoridine derivative 6j inhibits liver cancer cell proliferation via ATF3 mediated ferroptosis
Source: Cell Death Discov. 2023 Aug 14;9:296. doi: 10.1038/s41420-023-01597-6 (PMC10425377; doi:10.1038/s41420-023-01597-6)
Supplement: Supplementary file 1 — Figugre legends for Supplementary [file 41420_2023_1597_MOESM1_ESM.docx]

**Figure legends**

**Fig. S1 Sophoridine inhibited liver cancer cell proliferation in a ferroptosis independent manner.** (A) Sophoridine inhibited HepG2 and MHCC-97H cell proliferation in a dose-dependent manner. (B) The proliferation inhibition induced by sophoridine is ferroptosis independent.

**Fig. S2 ATF3 is widely expressed in human normal tissues**.
