# Supplementary figures and images for: Sophoridine derivative 6j inhibits liver cancer cell proliferation via ATF3 mediated ferroptosis
Source: Cell Death Discov. 2023 Aug 14;9:296. doi: 10.1038/s41420-023-01597-6 (PMC10425377; doi:10.1038/s41420-023-01597-6)

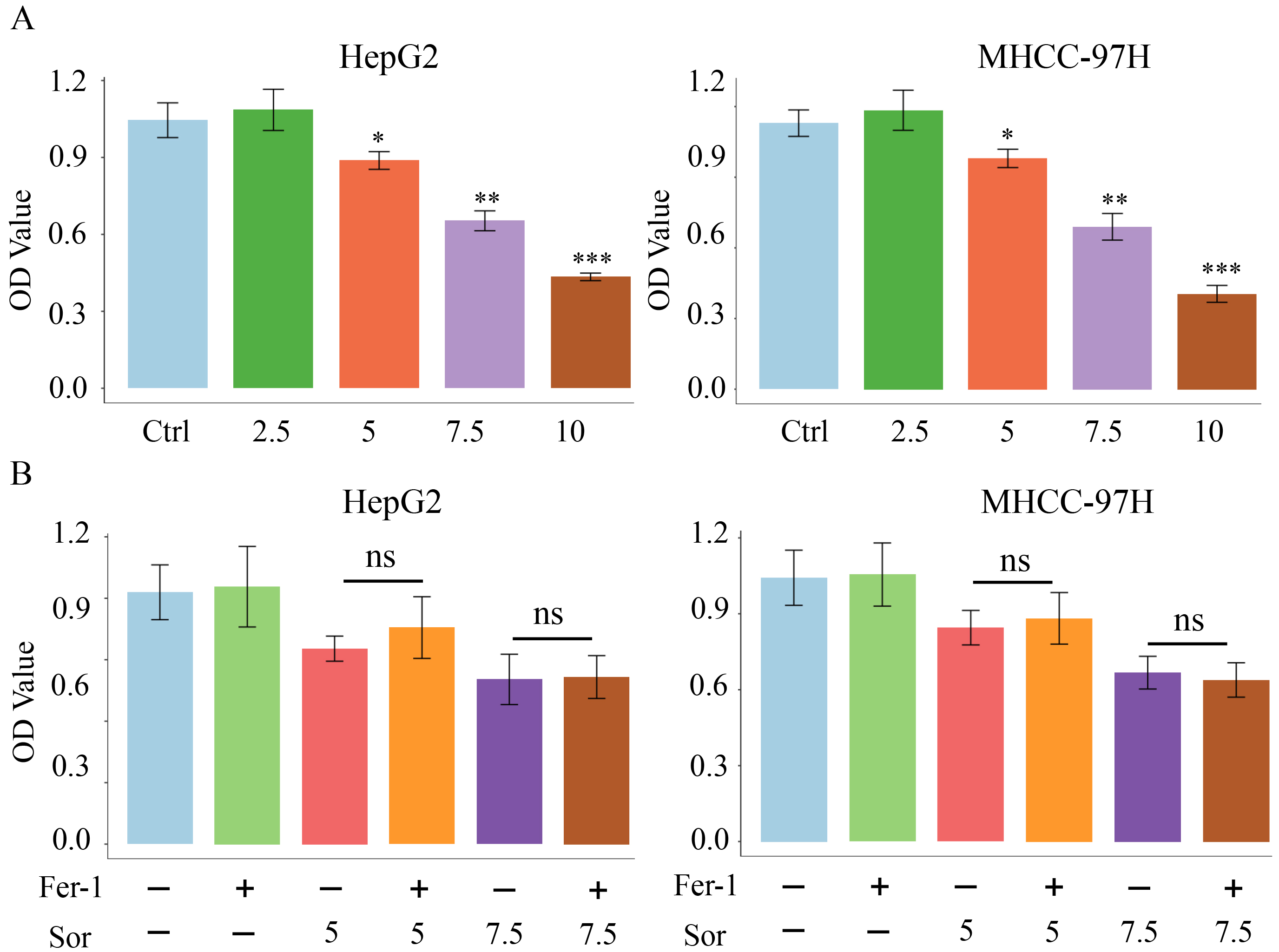

Supplement: Supplementary file 2 — Figure S1 [file 41420_2023_1597_MOESM2_ESM.jpg]

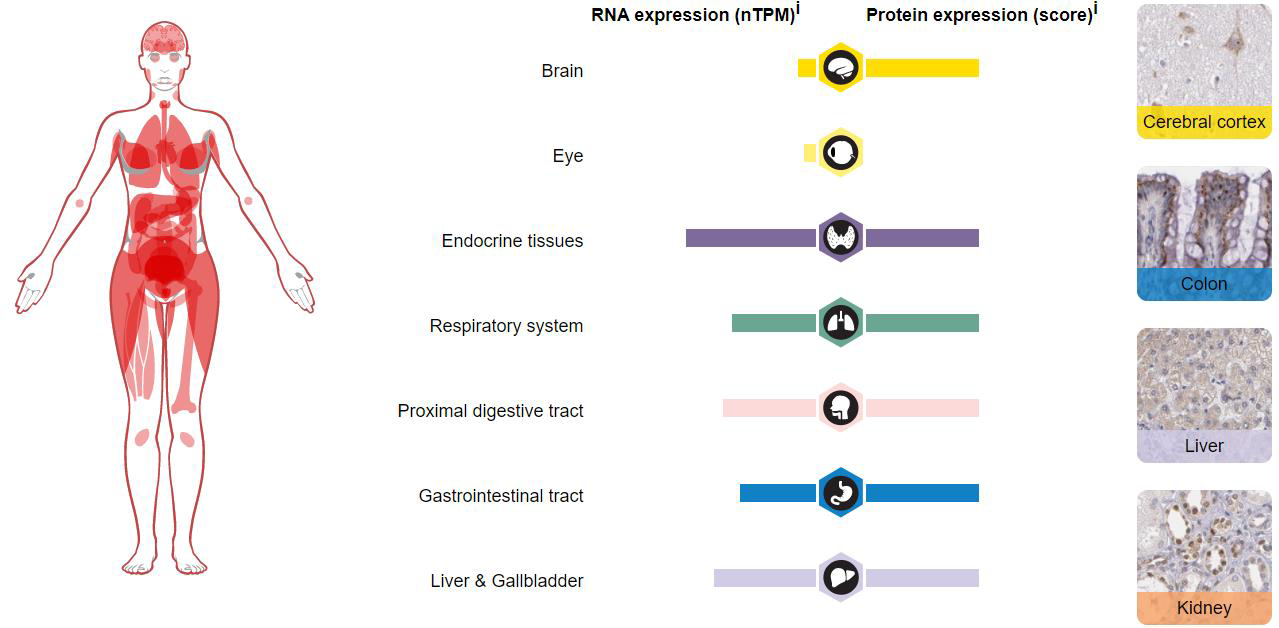

Supplement: Supplementary file 3 — Figure S2 [file 41420_2023_1597_MOESM3_ESM.png]
