# Supplementary material for: Sophoridine derivative 6j inhibits liver cancer cell proliferation via ATF3 mediated ferroptosis
Source: Cell Death Discov. 2023 Aug 14;9:296. doi: 10.1038/s41420-023-01597-6 (PMC10425377; doi:10.1038/s41420-023-01597-6)
Supplement: Supplementary file 4 — Full and uncropped western blots [file 41420_2023_1597_MOESM4_ESM.pptx]

## Slide 1
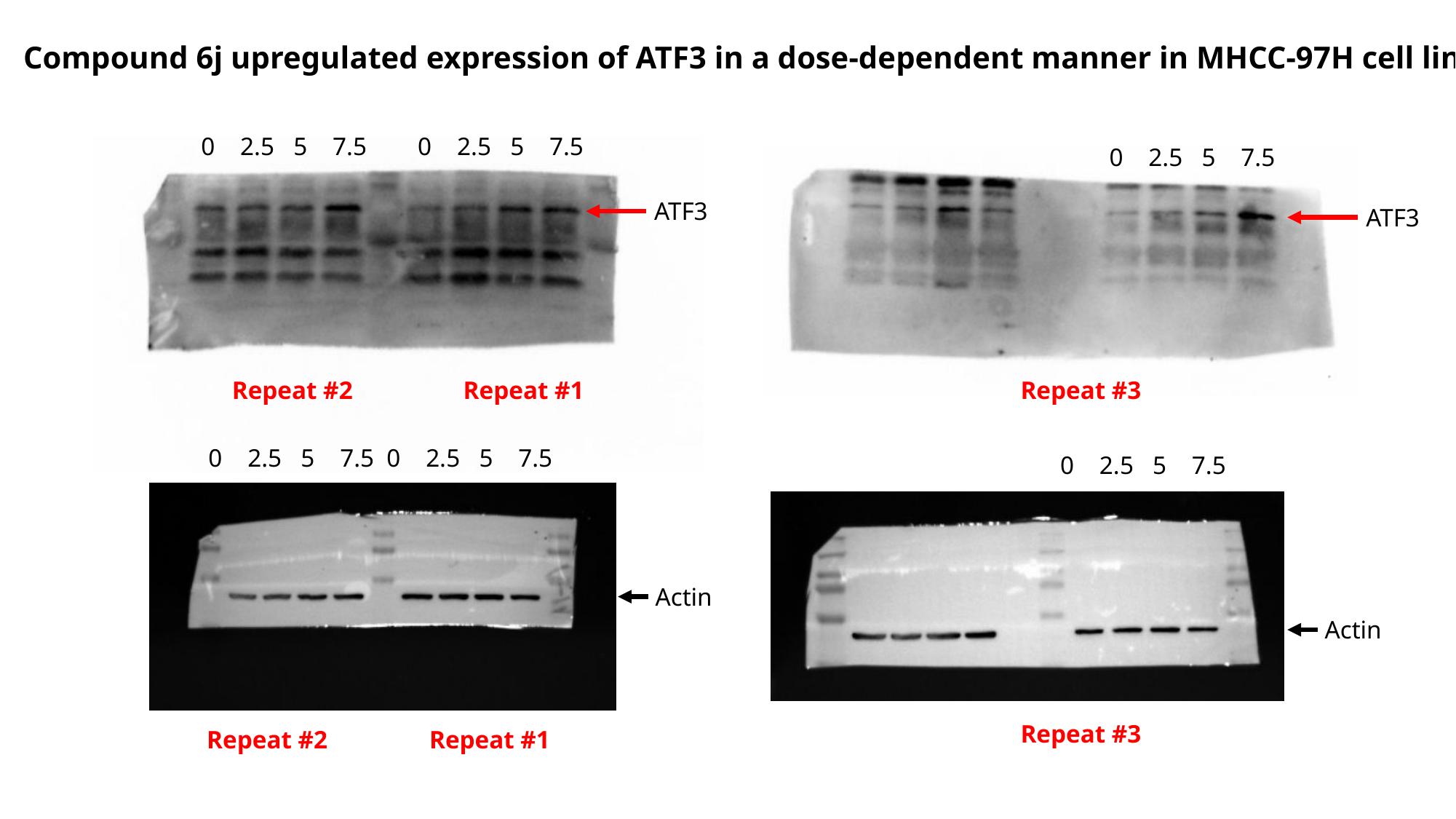

Compound 6j upregulated expression of ATF3 in a dose-dependent manner in MHCC-97H cell line
0 2.5 5 7.5
0 2.5 5 7.5
0 2.5 5 7.5
ATF3
ATF3
Repeat #2
Repeat #1
Repeat #3
0 2.5 5 7.5
0 2.5 5 7.5
0 2.5 5 7.5
Actin
Actin
Repeat #3
Repeat #2
Repeat #1

## Slide 2
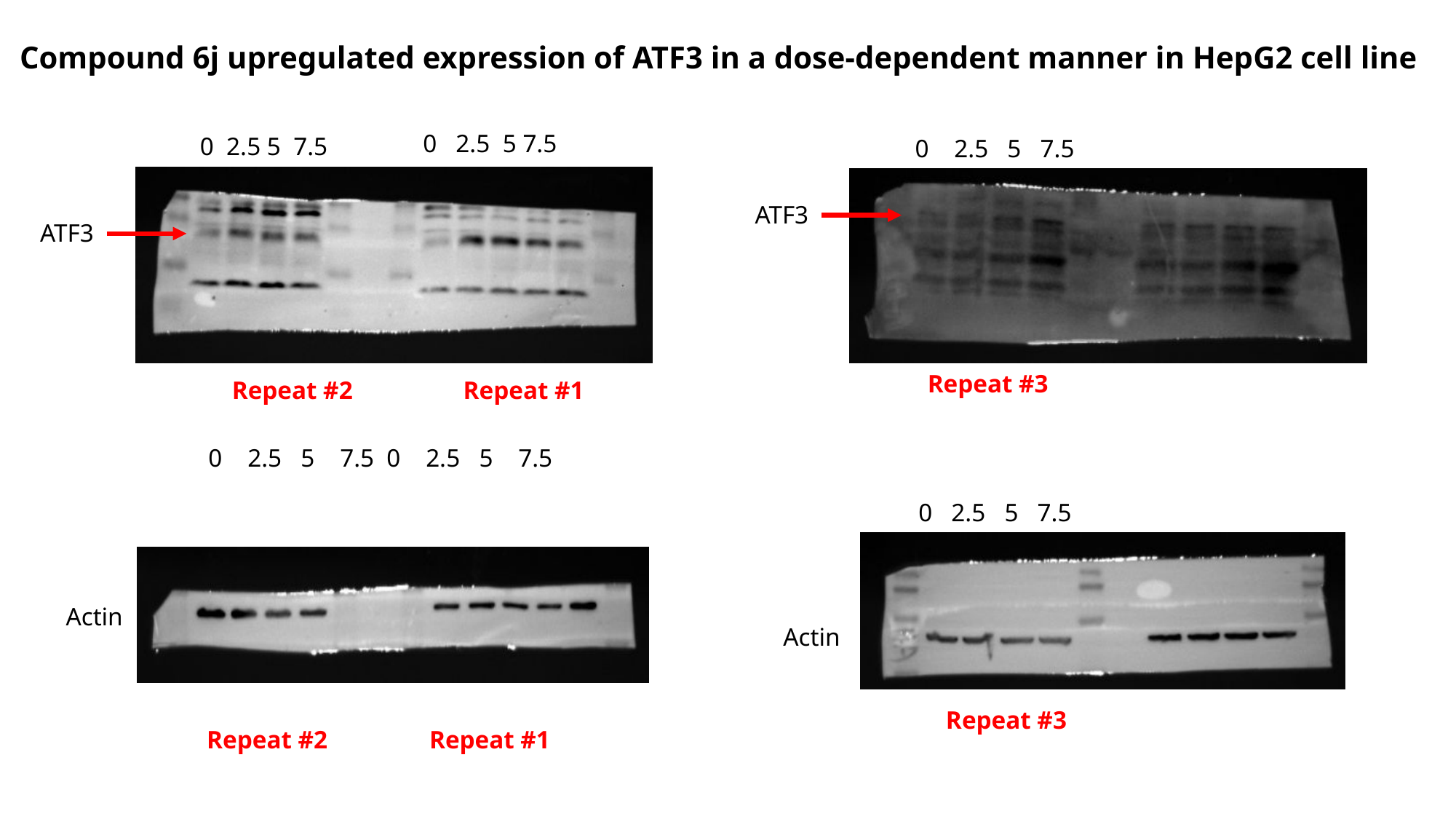

Compound 6j upregulated expression of ATF3 in a dose-dependent manner in HepG2 cell line
0 2.5 5 7.5
0 2.5 5 7.5
0 2.5 5 7.5
ATF3
ATF3
Repeat #3
Repeat #2
Repeat #1
0 2.5 5 7.5
0 2.5 5 7.5
0 2.5 5 7.5
Actin
Actin
Repeat #3
Repeat #2
Repeat #1

## Slide 3
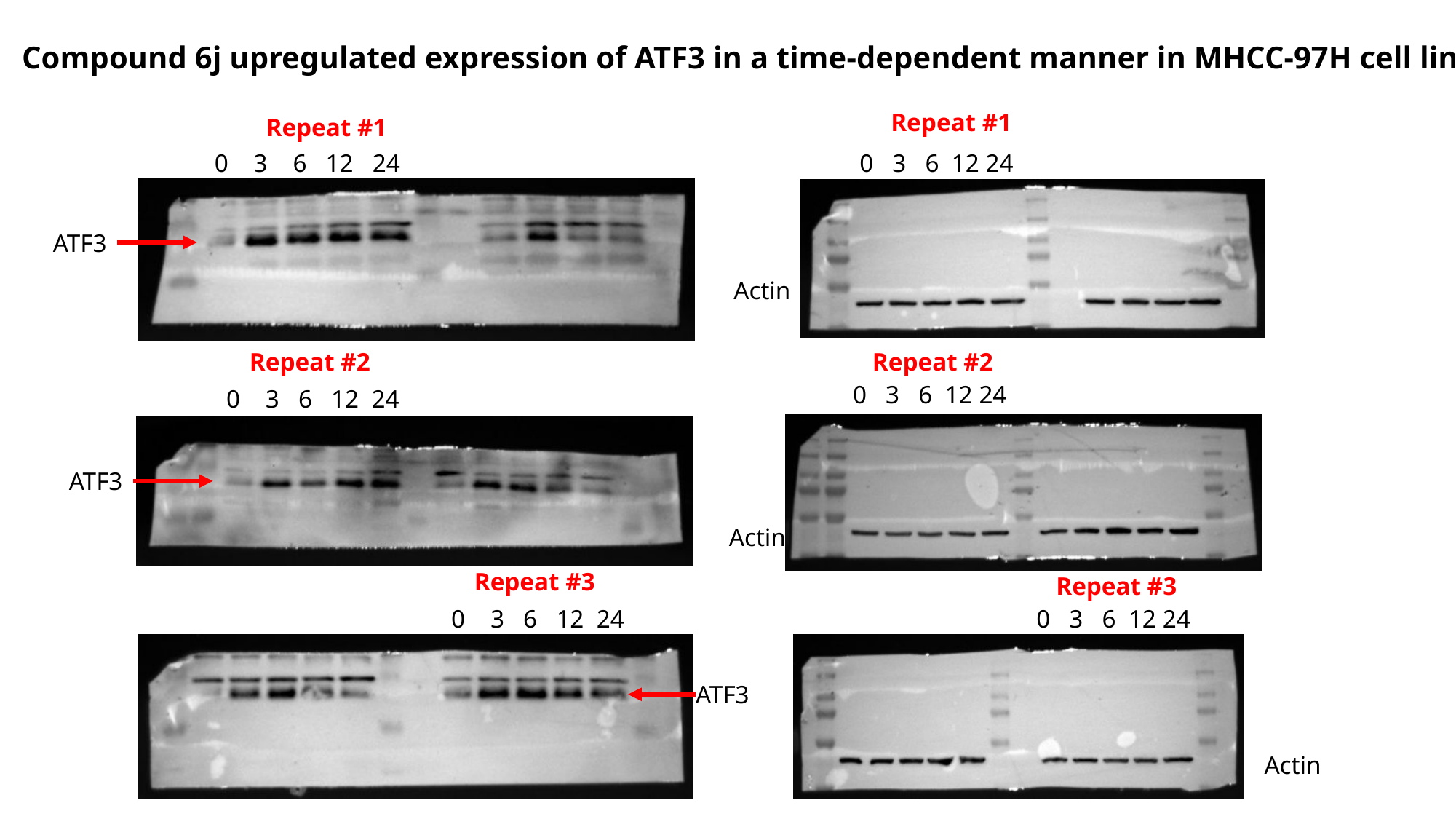

Compound 6j upregulated expression of ATF3 in a time-dependent manner in MHCC-97H cell line
Repeat #1
Repeat #1
0 3 6 12 24
0 3 6 12 24
ATF3
Actin
Repeat #2
Repeat #2
0 3 6 12 24
0 3 6 12 24
ATF3
Actin
Repeat #3
Repeat #3
0 3 6 12 24
0 3 6 12 24
ATF3
Actin

## Slide 4
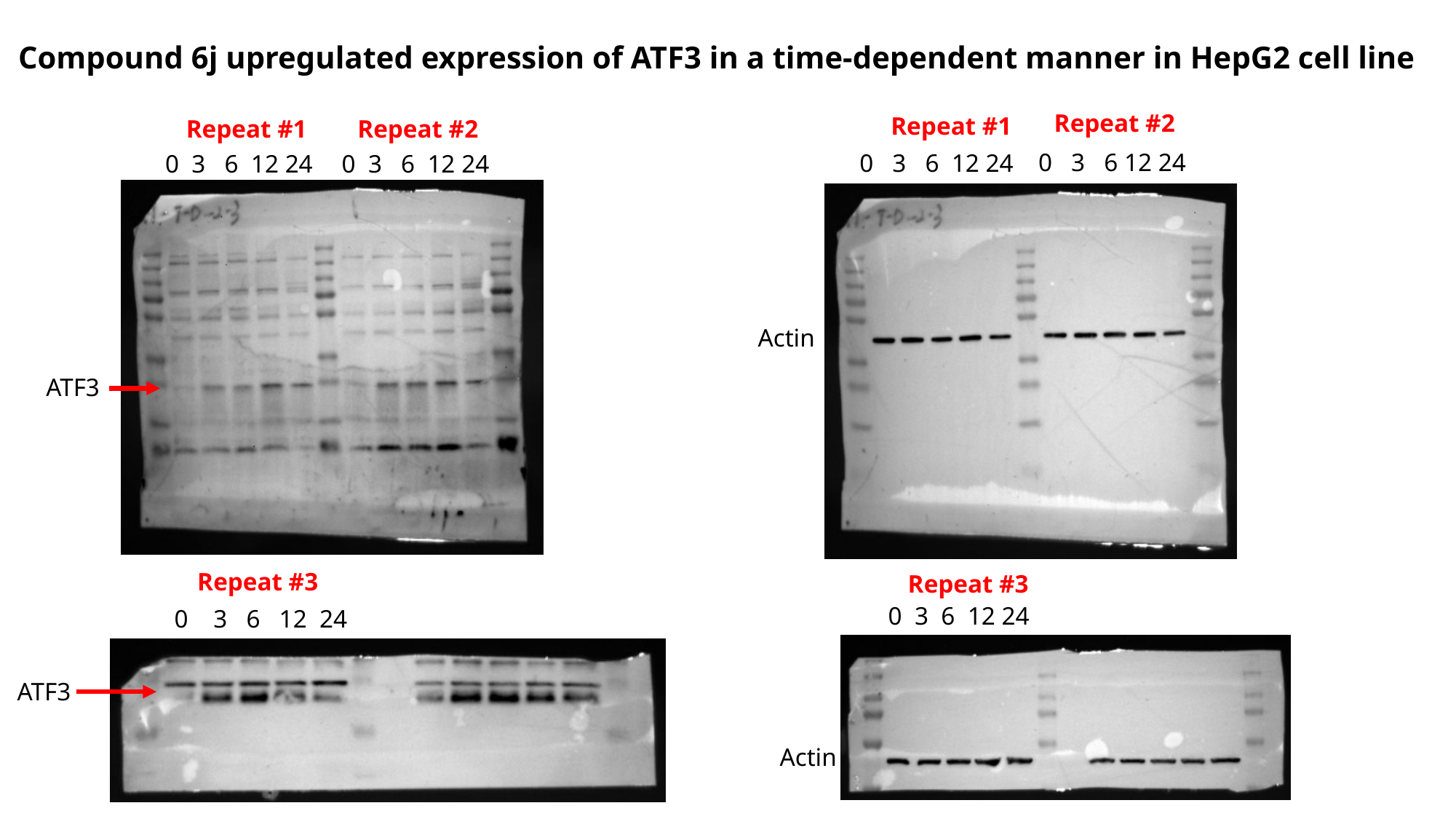

Compound 6j upregulated expression of ATF3 in a time-dependent manner in HepG2 cell line
Repeat #2
Repeat #1
Repeat #1
Repeat #2
0 3 6 12 24
0 3 6 12 24
0 3 6 12 24
0 3 6 12 24
Actin
ATF3
Repeat #3
Repeat #3
0 3 6 12 24
0 3 6 12 24
ATF3
Actin

## Slide 5
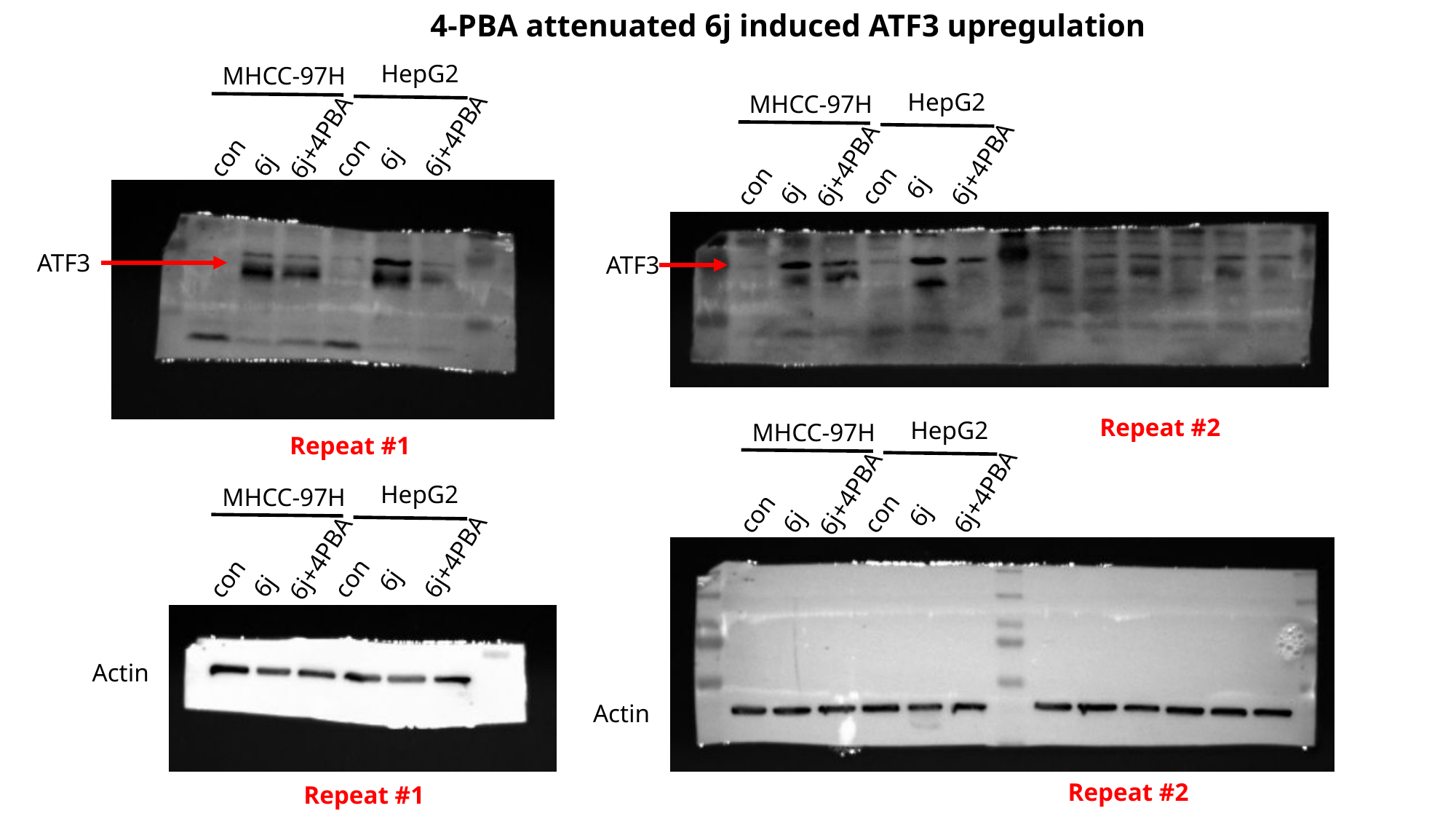

4-PBA attenuated 6j induced ATF3 upregulation
HepG2
MHCC-97H
HepG2
MHCC-97H
6j+4PBA
6j+4PBA
con
con
6j
6j+4PBA
6j
6j+4PBA
con
con
6j
6j
ATF3
ATF3
Repeat #2
HepG2
MHCC-97H
Repeat #1
6j+4PBA
6j+4PBA
HepG2
MHCC-97H
con
con
6j
6j
6j+4PBA
6j+4PBA
con
con
6j
6j
Actin
Actin
Repeat #2
Repeat #1

## Slide 6
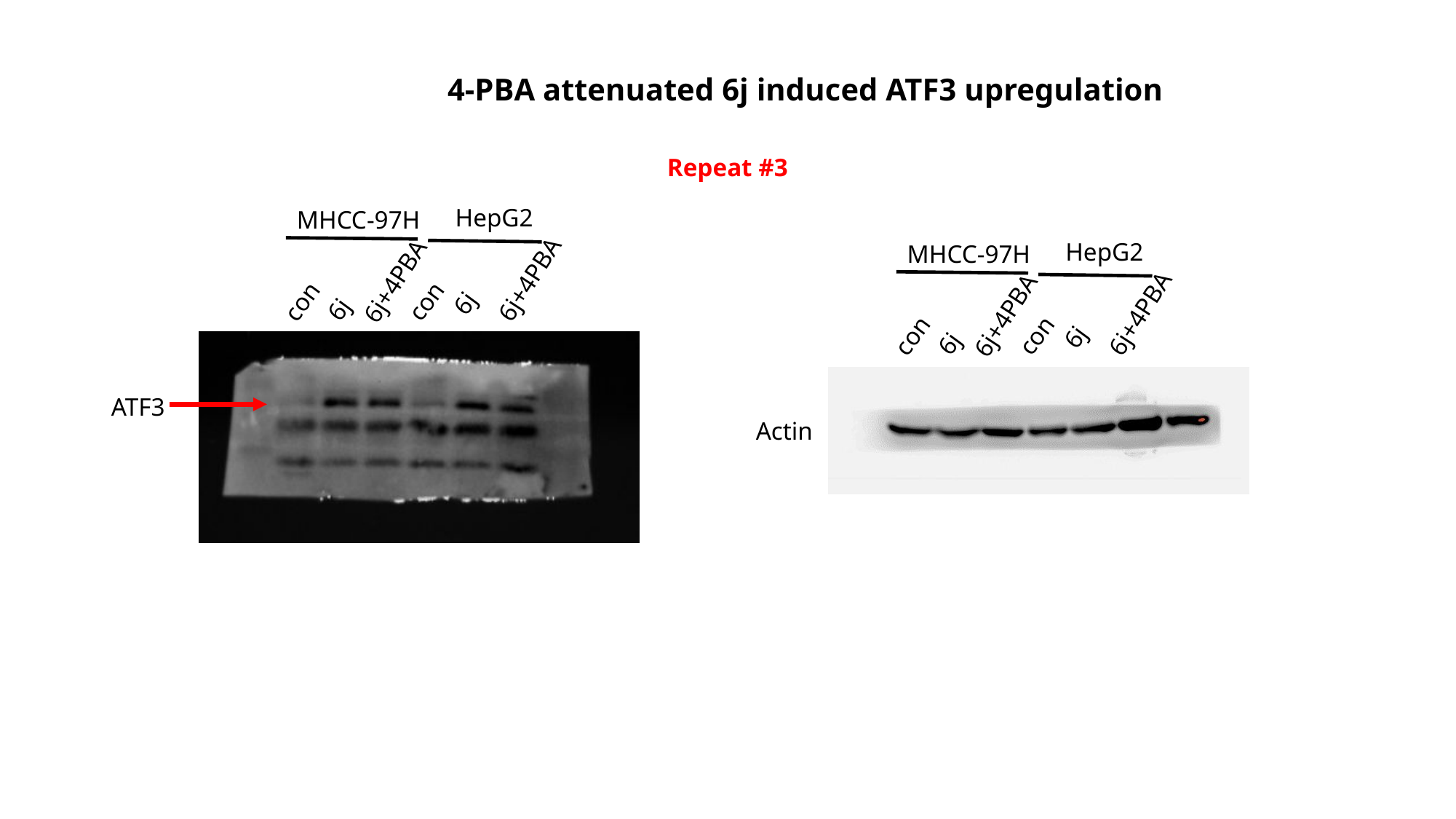

4-PBA attenuated 6j induced ATF3 upregulation
Repeat #3
HepG2
MHCC-97H
HepG2
MHCC-97H
6j+4PBA
6j+4PBA
con
con
6j
6j
6j+4PBA
6j+4PBA
con
con
6j
6j
ATF3
Actin

## Slide 7
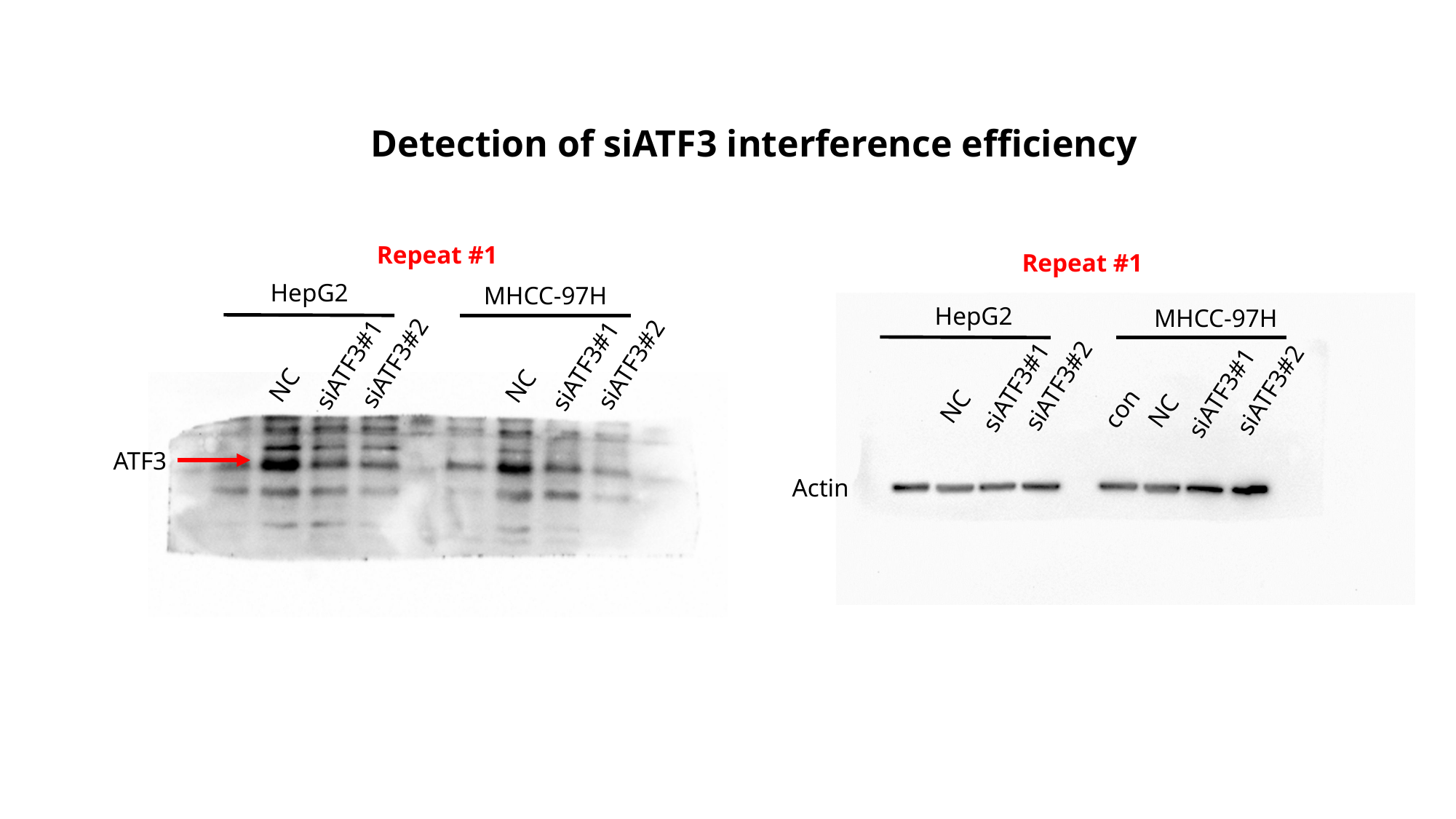

Detection of siATF3 interference efficiency
Repeat #1
Repeat #1
HepG2
MHCC-97H
HepG2
MHCC-97H
siATF3#2
siATF3#1
siATF3#2
siATF3#1
NC
siATF3#2
NC
siATF3#1
siATF3#2
siATF3#1
NC
con
NC
ATF3
Actin

## Slide 8
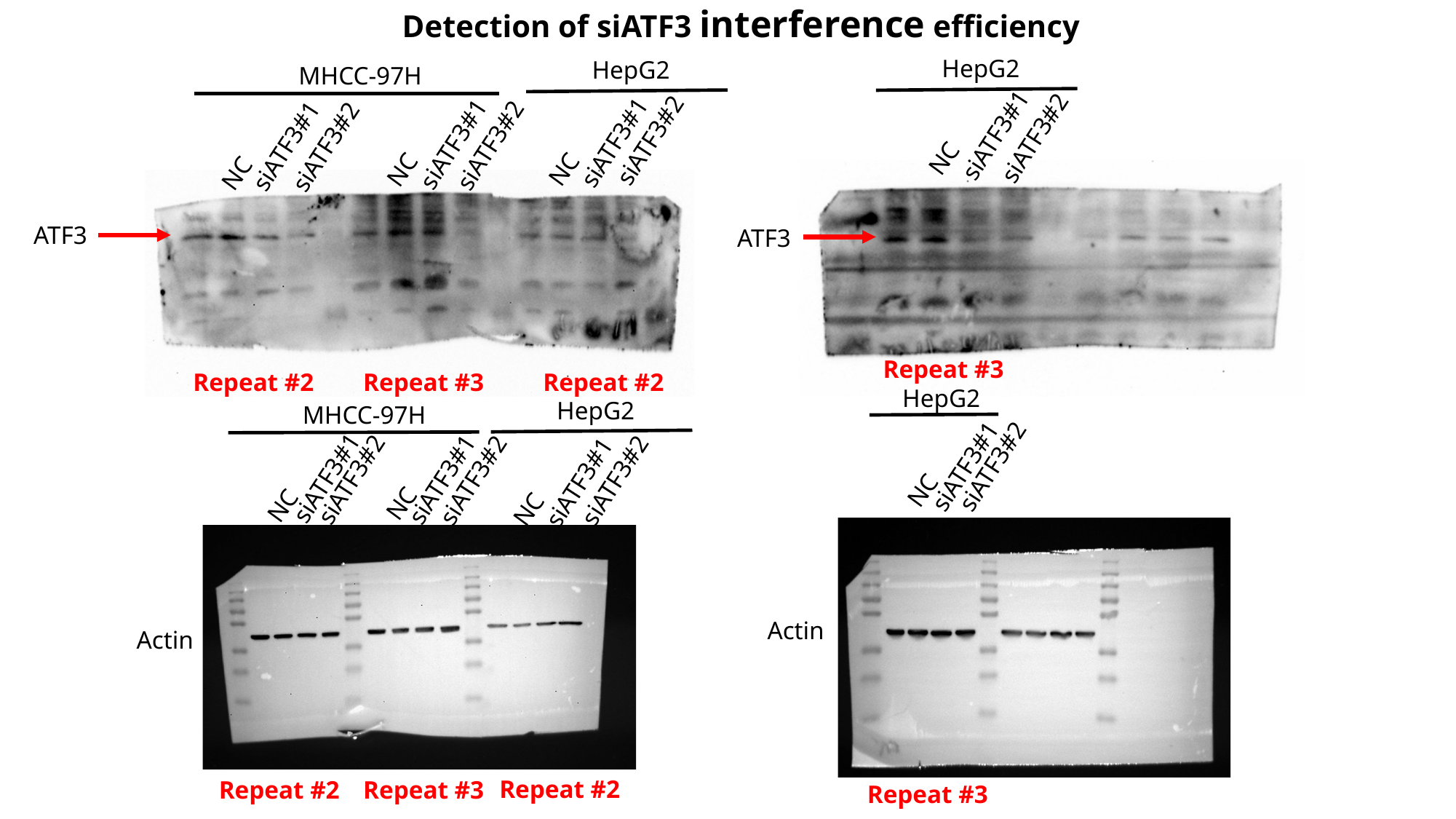

Detection of siATF3 interference efficiency
HepG2
HepG2
MHCC-97H
siATF3#1
siATF3#2
siATF3#2
siATF3#1
siATF3#1
siATF3#2
siATF3#1
siATF3#2
NC
NC
NC
NC
ATF3
ATF3
Repeat #3
Repeat #3
Repeat #2
Repeat #2
HepG2
HepG2
MHCC-97H
siATF3#1
siATF3#2
siATF3#1
siATF3#2
siATF3#1
siATF3#2
siATF3#2
siATF3#1
NC
NC
NC
NC
Actin
Actin
Repeat #2
Repeat #2
Repeat #3
Repeat #3

## Slide 9
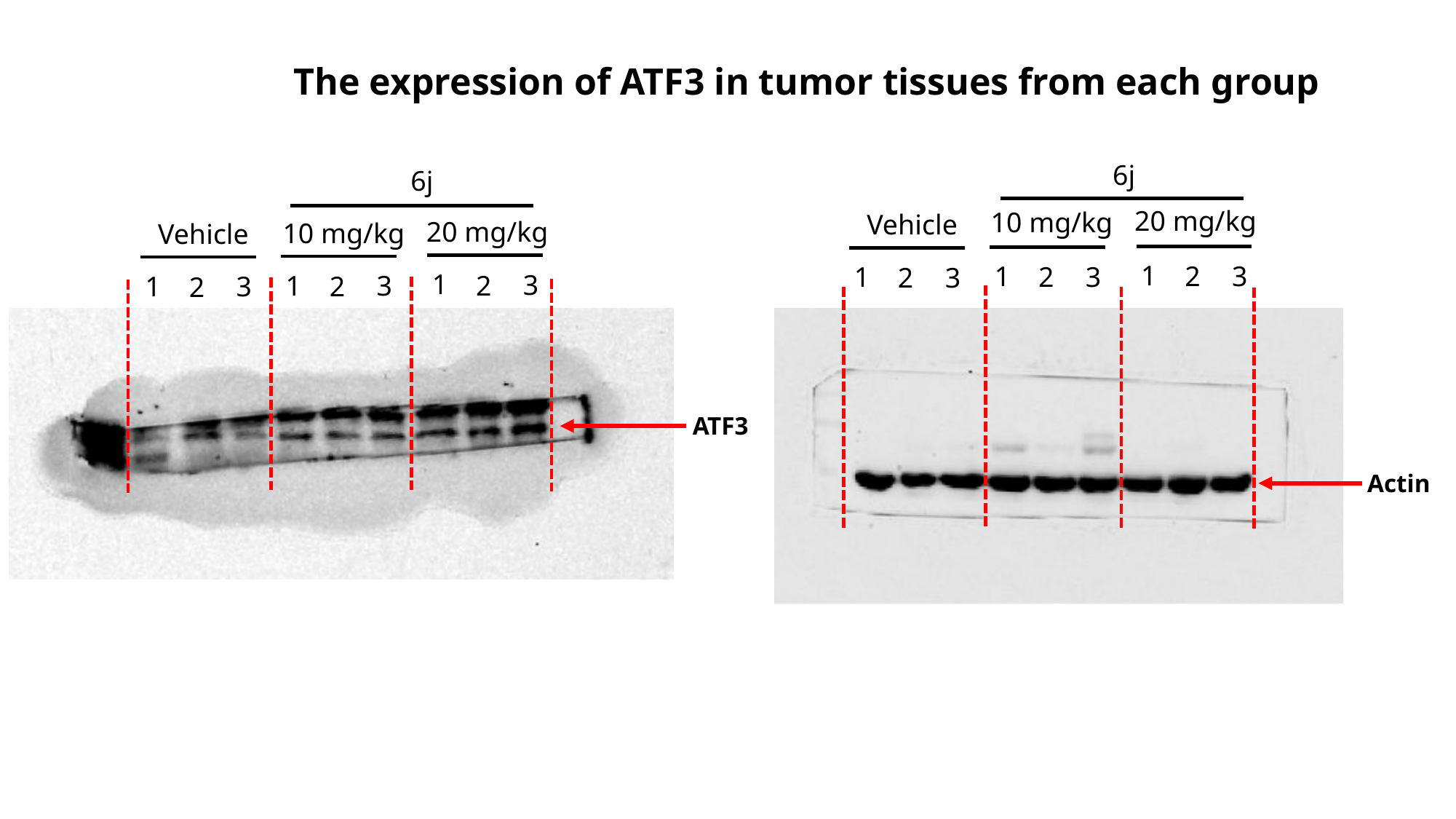

The expression of ATF3 in tumor tissues from each group
6j
6j
20 mg/kg
10 mg/kg
Vehicle
20 mg/kg
10 mg/kg
Vehicle
1
3
1
2
3
1
2
3
2
1
3
1
2
3
1
2
3
2
ATF3
Actin
